# Therapeutic Use of Acetaminophen and Light to Moderate Alcohol: Are There Early Disparate Risks for Kidney Disease?

**DOI:** 10.1089/heq.2020.0072

**Published:** 2020-12-14

**Authors:** Harrison Ndetan, Marion W. Evans, Terence Tanue, Christie C. Osuagwu, Emmanuel Elueze, Karan P. Singh, Kirk Calhoun

**Affiliations:** ^1^ Department of Epidemiology and Biostatistics, School of Community and Rural Health, University of Texas Health Science Center at Tyler, Tyler, Texas, USA.; ^2^ Department of Food Science, Nutrition, and Health Promotion, Mississippi State University, Mississippi State, Mississippi, USA.; ^3^ Data Analytics, Genpact LLC, Jacksonville, Florida, USA.; ^4^ Department of Community Health, School of Community and Rural Health, University of Texas Health Science Center at Tyler, Tyler, Texas, USA.; ^5^ Medical Education and Professional Development, University of Texas Health Science Center at Tyler, Tyler, Texas, USA.; ^6^ Department of Medicine, University of Texas Health Science Center at Tyler, Tyler, Texas, USA.

**Keywords:** drug safety, renal disease, alcohol misuse, health disparities, acetaminophen

## Abstract

**Introduction:** Acetaminophen (APAP) is the most common medication taken in the United States. Using the 2003–2004 U.S. National Health and Nutrition Examination Survey (NHANES), the authors previously explored and reported the relationship of concomitant consumption of light to moderate alcohol (LMA) and therapeutic doses of APAP to early risk of renal dysfunction among adults in the United States. Statistically significant increased odds of renal dysfunction were noted among respondents who reported use of therapeutic doses of APAP and LMA by adjusting for hypertension, diabetes, and obesity. In this study the authors explored further on of potential disparities in the above relationship in the population. The relationship was verified with the 2009 Chronic Kidney Disease–Epidemiology Collaboration creatine-based estimated Glomerular Filtration Rate (GFR).

**Methods:** Subgroup logistic regression analyses to assess disparities based on gender, race, age, education, and income were performed for renal dysfunction measured in terms of serum creatinine (SCr) directly as well as self-report using NHANES 2003–2004 data.

**Results:** Early stage renal dysfunction, as determined by self-reports, and SCr and GFR values may occur among those who concomitantly ingested therapeutic doses of APAP and described alcohol use when compared to those who do not. Risks were more profound among females, particularly in minority racial groups, below legal drinking age of 21, and with household income below $25K.

**Conclusion:** Potential risks for renal dysfunction are apparent in a disparate manner resulting in possible health inequity. Further research could increase the sample size of minority groups and specifically assess for effect modifiers that NHANES does not include for assessment.

## Introduction

According to the National Survey on Drug Use and Health,^1^ the lifetime prevalence of alcohol use in the 18 and older age group is 86.3% within the United States. Further, acetaminophen (APAP) is a common pain reliever in hundreds of over-the-counter and prescription medications.^2^ According to the Consumer Healthcare Products Association (CHPA), APAP is the most common drug ingredient in the United States.^3^ The CHPA states that 52 million U.S. consumers use APAP containing medications each week, which is ∼23% of the population. APAP use has been associated with hepatotoxicity, including frank liver failure when taken in doses greater than the recommended limit of 4 g per day.^4–6^ Renal injury from use of APAP at toxic levels or in conjunction with alcohol has been reported as well.^7–10^

In our previous article, we reported on concurrent use of light to moderate alcohol (LMA) and consumption of recommended therapeutic doses of APAP and the risk of kidney dysfunction through an evaluation of the National Health and Nutrition Examination Survey (NHANES).^11^ This article reports on further examination of those data regarding disparities related to those risks. Specifically, we aimed to assess the risk of early self-reported kidney disease through data analysis of the NHANES from 2003 to 2004 when the survey was accompanied by various blood chemistries that included kidney biomarkers. The NHANES is a series of cross-sectional and national noninstitutionalized representative surveys conducted through a program of studies designed by National Center for Health Statistics of the Centers for Disease Control and Prevention to assess the health and nutritional status of adults and children in the United States.^12^ A specific focus on gender, race, age, education, and income were assessed for effects.

## Methods

This study was provided an expedited Institutional Review Board approval due to it being secondary data analysis.

The study is a secondary data analysis of the 2003–2004 NHANES dataset, which included over-the-counter medicine usage. Details of the data sources, multistage probability design sampling, and the analytical sample have been previously reported by us.^11^ Variables for this analysis were extracted from the questionnaire (interview), medical examination, and laboratory data files.

### Measures

The outcome variable was kidney function defined separately based on self-reported survey responses, and laboratory-based kidney biomarkers and function tests. Affirmative responses to survey questions that asked questions such as whether or not respondents were ever told they had “weak/failing kidneys,” and “received dialysis in past 12 months,” or self-reports of abnormal kidney function all constituted “renal dysfunction” as well as serum creatinine (SCr) >1.0 mg/dL, and glomerular filtration rate (GFR) <90 mL/min/1.73 m^2^.

GFR was estimated using the 2009 Chronic Kidney Disease–Epidemiology Collaboration (CKD-EPI) creatine-based prediction equation^13–15^:

$$\begin{array}{c} GFR=141\times min{\left(SCr∕\kappa ,1\right)}^{\alpha }\times max{\left(SCr∕\kappa ,1\right)}^{-1.209}\\ \times 0.993^{Age}\times 1.018\left[iffemale\right]\times 1.159\left[ifBlack\right] \end{array}$$

where κ=0.7 (females) or 0.9 (males), α=−0.329 (females) or −0.411 (males), min=indicates the minimum of SCr/κ or 1, max=indicates the maximum of SCr/κ or 1, and age in years.

This equation has been touted as being more accurate in estimating GFR and prognosis than the 2006 Modification of Diet in Renal Disease Study equation and used in assessing GFR in diverse populations.^13–14^

#### APAP exposure

In NHANES 2003–2004, participants were asked whether they had, “used pain relievers in the past 30 days,” followed by a list of pain-relievers, including APAP/APAP-containing products. Respondents also reported the strength of the medication they were using as stated on the drug package. Exposure to therapeutic APAP was defined as “Yes” for taking not more than 6 pills or 4000 mg of APAP/APAP-containing product in a day on a regular basis and “No” if they reported not taking any.

#### Alcohol exposure

Survey questions queried respondents on how often they drank alcohol over past 12 months and the average number of alcoholic drinks they consumed per day. A drink was defined as 12 oz of beer, a 4 oz glass of wine, or an ounce of liquor. Consumption of up to one drink per day for women and up to two drinks per day for men constituted exposure to light/moderate amount of alcohol with no alcohol as the comparative group. Those who consumed more than these limits were considered heavy drinkers and were excluded from the analysis.

#### Sociodemographics and potential predisposing factors

This report focuses on disparities in the relationship of early stage renal dysfunction potentially associated with the use of therapeutic amount of APAP and LMA consumption based on gender, race/ethnicity, age, education, and income while controlling for predisposing factors, including hypertension, obesity, and diabetes.

The racial subgroups considered were white, blacks, Hispanics, and others. Age that was originally captured in scale was further categorized into “<21,” “21–30,” “30+ to <65,” and “≥65”; education grouped into “High school and below”; “High school graduates and some college;” and “College graduate” and income into “<$25,000,” “$25,000 to <$75,000,” and “≥$75,000.”

During medical examination, three measurements of resting blood pressure (BP) were recorded for each participant. Hypertension was noted if the averages of last two readings of systolic BP was at least 130 mmHg or diastolic BP was at least 80 mmHg (American College of Cardiology, 2017) or whether a doctor had ever told the participant he/she had hypertension, or they reported taking high BP medication. Obesity was defined by body mass index >30 kg/m^2^ and diabetes by plasma glucose levels >126 mg/dL.

### Statistical analysis

Data analyses were performed using Statistical Analysis System (SAS), version 9.4 (Cary, NC) software. The complete multistage probability design structure with appropriate differential selection probabilities (medical examination and laboratory data, MEC weight) and geographic clustering/stratification (survey cluster/strata) was applied to generate unbiased national population estimates (NPEs) of the United States and weighted percentages (%) for each variable, other parameters, and variance estimations.

Odds ratios (OR) and corresponding 95% confidence intervals (CI) that assessed the likelihood that individuals exposed to therapeutic amounts of APAP and/or light to moderate amount of alcohol would have renal dysfunction compared to those not similarly exposed were obtained using binary logistic regression models generated for various gender, racial, age, educational, and income subgroups. Comorbidities such as diabetes, hypertension, and obesity, that could predispose the kidney to APAP toxicity,^15–17^ were controlled for using multiple logistic regression models, generating adjusted odds ratios (AOR) and the corresponding 95% CI. Subgroup analyses to assess disparities based on gender, race, age, education, and income were performed and reported only for renal dysfunction measured in terms of SCr directly, as estimated GFR already made considerations of physiological contributions of muscle mass based on gender, age, and race.^13–15,18–20^

## Results

A general description of the analytical sample was previously reported by Ndetan and others, in 2018. In brief, and for the purposes of this analysis, females made up 51% of the sample. Almost 69% were white, 12% blacks, and 13% Hispanics. About 30% were less than the legal drinking age of 21 years and 12% were 65 years old or more. More than 58% had high school or some college education, and 23% were college graduates. About 48% of the sample fell into the income category of $25,000–75,000 annually, with 27% earning ≥$75,000. The overall NPE for self-reported renal dysfunction (SRRD) was 65,223,770 (32%), and NPE for elevated SCr was 71,066,136 (25%) when calculated at ≥1.0 mg/dL. Table 1 shows the distribution (NPE, %) of those with SRRD and elevated SCr based on gender, race, age, education, and income only for those reporting these variables.

**Table 1.** Prevalence [National Population Estimates (Weighted Percent)] of Renal Dysfunction Among Those Who Ingested Therapeutic Dose of Acetaminophen and Light-Moderate Amount of Alcohol by Sociodemographic Characteristics (National Health and Nutrition Examination Survey 2003–2004)

	Self-report renal dysfunction	SCr ≥1.0mg/dL	GFR <90.0 mL/min/1.73 m^2^
	NPE=9,062,965	NPE=8,890,441	NPE=9,578,520
Gender			
Female	7,242,219 (79.91)	2,663,860 (29.96)	3,385,308 (35.34)
Male	1,820,746 (20.09)	6,226,581 (70.04)	6,193,213 (64.66)
Race			
White	7,593,596 (83.79)	7,147,254 (80.39)	8,016,310 (83.69)
Black	829,997 (9.16)	770,397 (8.67)	470,929 (4.92)
Hispanic	401,880 (4.43)	335,050 (3.77)	359,641 (3.75)
Other	237,492 (2.62)	637,741 (7.17)	731,640 (7.64)
Age (years)			
<21	—	219,169 (2.47)	—
21–30	544,046 (6.00)	715,869 (8.05)	456,019 (4.76)
>30 to <65	2857987 (31.53)	2,218,554 (24.95)	2,407,086 (25.13)
≥65	5,660,933 (62.46)	5,736,850 (64.53)	6,715,416 (70.11)
Education			
Less than high school	1,592,287 (17.57)	1,332,719 (15.41)	1,496,286 (15.66)
High school to college	4,985,647 (55.01)	5,017,021 (58.02)	5,693,443 (59.59)
College graduate	2,485,031 (27.42)	2,298,038 (26.57)	2,365,297 (24.75)
Household income			
<25K	2,520,896 (29.89)	2,328,976 (27.88)	2,398,009 (26.52)
25K to <75K	3,966,475 (47.03)	4,682,805 (56.05)	5,168,883 (57.17)
≥75K	1,945,999 (23.08)	1,342,753 (16.07)	1,474,277 (16.31)

GFR, glomerular filtration rate; NPE, national population estimates; SCr, serum creatinine.

### Self-reported renal dysfunction

Within the sample, almost 80% of those who reported light to moderate drinking and therapeutic APAP use along with SRRD were female. Over 83% were white, and almost 64.5% were 65 years of age or older. Fifty-five percent with a high school education or some college had SRRD and 47% of those with an income in the range of $25,000–75,000. That income group represented the majority of those reporting (Table 1).

### Overall assessment of risk of renal dysfunction

Using the estimated GFR, there was an overall statistically significant increased likelihood of early stage kidney dysfunction in those using LMA and therapeutic doses of APAP compared to those who did not (OR=3.22, 95% CI=2.45–4.24; AOR=2.65, 95% CI=1.72–4.09).

### Disparate renal dysfunction

Table 2 depicts estimated crude and adjusted effected measures (OR and 95% CI) for renal dysfunction measured by elevated SCr potentially associated with therapeutic dose of APAP and light to moderate drinking.

**Table 2.** Relationships (Odds Ratio and 95% Confidence Intervals) of Renal Dysfunction to Therapeutic Dose of Acetaminophen and/or Light/Moderate Amount of Alcohol Among Various Gender, Racial, and Age Groups (National Health and Nutrition Examination Survey 2003–2004)

Sociodemographic characteristics	Crude		Adjusted^a^	
	Self-report renal dysfunction	SCr ≥1.0 mg/dL	Self-report renal dysfunction	SCr ≥1.0 mg/dL
Gender				
Female	1.90 (1.33–2.70)	4.20 (2.88–6.12)	1.87 (1.19–2.95)	0.85 (0.36–2.01)
Male	1.37 (0.92–2.05)	2.20 (1.63–2.97)	1.19 (0.46–3.08)	1.59 (0.96–2.64)
Race				
White	1.63 (1.23–2.16)	1.90 (1.51–2.39)	1.87 (1.14–3.08)	0.80 (0.39–1.62)
Black	2.27 (1.45–3.54)	2.13 (1.29–3.53)	1.05 (0.22–5.08)	2.40 (0.79–7.32)
Hispanic	1.50 (0.71–3.16)	2.91 (1.23–6.93)	1.04 (0.23–4.75)	0.61 (0.06–6.17)
Other	0.84 (0.19–3.68)	5.82 (1.83–18.51)	3.63 (0.20–64.84)	22.26 (1.15–328.63)
Age (years)				
<21	—	11.21 (4.74–26.52)	—	9.01 (1.88–43.13)
21–30	3.20 (0.68–15.10)	1.28 (0.28–5.78)	7.52 (2.00–28.29)	0.37 (0.04–3.21)
>+30 to <65	1.19 (0.84–1.69)	1.13 (0.81–1.57)	1.35 (0.88–2.07)	1.26 (0.80–1.98)
≥65	1.00 (0.69–1.45)	1.11 (0.83–1.49	1.01 (0.59–1.73)	1.40 (0.89–2.22)
Education				
High school and below	1.49 (0.99–2.25)	1.43 (0.96–2.14)	0.90 (0.44–1.81)	0.70 (0.41–1.20)
High graduate and some college	1.68 (1.22–2.33)	1.60 (1.24–2.07)	1.87 (1.16–3.01)	1.78 (1.14–2.80)
College graduate	1.73 (0.99–3.02)	1.08 (0.65–1.80)	1.57 (0.62–3.95)	1.23 (0.44–3.41)
Household income				
<25K	1.50 (0.94–2.40)	3.03 (2.14–4.29)	1.44 (0.74–2.81)	1.91 (1.00–3.65)
25 to <75K	1.37 (0.97–1.95)	2.19 (1.50–3.20)	1.25 (0.66–2.36)	1.50 (0.92–2.42)
≥75K	2.39 (1.27–4.50)	1.44 (0.90–2.32)	2.34 (0.99–5.53)	1.11 (0.45–2.76)
≥25K	1.65 (1.18–2.30)	1.95 (1.46–2.59)	1.49 (0.94–2.38)	1.33 (0.89–1.98)

eGFR (based on the 2009 Chronic Kidney Disease–Epidemiology Collaboration equation): Crude OR (95% CI)=3.22 (2.45–4.24); adjusted: 2.65 (1.72–4.09). This will go in results text.

^a^ Adjusted for hypertension, obesity, and diabetes.eGFR, estimated glomerular filtration rate.

#### Gender

Female SRRD was significantly greater (OR=1.90; 95% CI=1.33–2.70). Females were also more likely to have abnormal SCr (OR=4.20; 95% CI=2.88–6.12), and SRRD was maintained in the adjusted model (OR=1.87; 95% CI=1.19–2.95). but SCr >1.0 mg/dL was not. Male users had an increased risk of SCr abnormalities (OR=2.20; 95% CI=1.63–2.97), but this was not significant in the adjusted model nor was in SRRD.

#### Race

Race categories were white, black, Hispanic, and “other.” Whites made up almost 84% of those with SRRD, 80% of those with SCr abnormalities, and 84% with GFR abnormalities. Whites' risk for SRRD was significant at an OR of 1.63 (95% CI=1.23–2.16) and SCr abnormality was as well (OR=1.90; 95% CI=1.51–2.39). For whites, the adjusted SRRD was still significant (OR=1.87; 95% CI=1.14–3.08). In blacks SRRD was significant (OR=2.27; 95% CI=1.45–3.54) as was SCr abnormalities (OR=2.13; 95% CI=1.23–6.93), but neither were maintained in an adjusted model. Hispanic participants had significance with crude SCr abnormalities (OR=5.82, 95% CI=1.83–18.51), but this was not significant in the adjusted model. “Other” indicated increased risk of SCr (OR=5.82, 95% CI=1.83–18.51) and with wide CIs in an adjusted model, SCr abnormality was maintained (OR=22.26; 95% CI=1.15–328.63).

#### Age

Those aged ≥65 years made up most of those with SRRD at 62.5%, and also 65% of those with SCr abnormalities. They also made up 70% of those with GFR abnormalities. In the <21 age category, sample numbers were small and only SCr abnormalities indicated significance in both the crude rates (OR=11.21; 95% CI=4.74–26.52) and adjusted (OR=9.01; 95% CI=1.88–43.13). For the 21–30 age range, only adjusted SRRD was significant (OR=7.52; 95% CI=2.00–28.29). No other age range had significance in either the crude calculations or adjusted.

#### Education

Overall, those in the high school and some college education category made up the highest percentages of those with SRRD (55%), SCr abnormalities (58%), and GFR abnormalities (59.6%). Interestingly, only one education category, “high school and some college,” showed significance, and this was maintained across both crude and adjusted assessments. For that category, risks for SRRD were as follows: Crude OR=1.68 (95% CI=1.22–2.33); SCr abnormality OR=1.60 (95% CI=1.24–2.07); and AOR for SRRD was 1.87 (95% CI=1.16–3.01) adjusted (OR=1.78; 95% CI=1.14–2.80).

#### Household income

Overall income levels indicated that those with SRRD were typically in the $25,000–75,000 income earning categories with that category making up 47% of those with SRRD. They also had the highest levels reported SCr abnormalities (56%) and GFR abnormalities (57%).

Household income in the $25,000 and under category was significantly associated with increases in risk of SCr abnormalities (Crude OR=3.03; 95% CI=2.14–4.29) but not maintained in an adjusted model. The same was true for SCr in the $25–75,000 range (OR=2.19; 95% CI=1.50–3.20) and this was not maintained in the adjusted model either. For an income variable calculated as ≥$25,0000, both the crude SRRD (OR=1.65; 95% CI=1.18–2.30) and SCr (OR=1.95; 95% CI=1.46–2.59) were significant, but neither held to significate in the adjusted model.

## Discussion

After controlling for hypertension, obesity, and diabetes, the concurrent use of what amounts to labeled use of APAP along with LMA use may be problematic for some people. Our earlier work using NHANES concluded this. As noted, APAP and drugs that contain it are among the most common consumed by Americans. Further, they are available over-the-counter in many formulations. The assessment performed here indicates that women may be at greater risk of renal dysfunction and the use of the common medications containing APAP along with what may be seen as harmless alcohol use could predispose them to greater risk. This risk may also be more relevant to those who are Hispanic or black compared with white. Even though whites made up more of the NHANES sample, the crude OR for blacks was 2.27 for SRRD and 21.3 for SCr abnormalities. This is compared to 1.63 for whites. The same can be said for Hispanics in the assessment of crude SCr in this assessment but not for SRRD. Even though wide CIs were noted for the “other” race category, those crude SCr risks (OR=5.82) and an AOR of 22.26 could indicate a notable risk in this group. Although poorly defined and low in numbers within the sample, the risk is detectible. In addition, we found it of interest that although self-reported kidney issues were not noted in the <21 age, irregularities in both crude and adjusted SCr levels were noted. This could indicate a risk that is currently unknown to many within this subpopulation. In addition, kidney comorbidities are more likely in minority groups.^21^ Health literacy could also be a complicating factor as well.^22^

An assessment using the 2000 and 2005 National Alcohol Surveys indicated that poverty ratios (white to black and Hispanic to white) were predictive of light to heavy drinking.^23^ However, income inequality was not. The authors suggested that higher levels of alcohol-related problems in higher income-equality states could be more related to social conditions. This could also be the case here, as the mid-income category was noted more significant than lower or higher categories. Education levels are equally perplexing in this assessment and could also be affected by social settings or conditions.

There are some limitations to this study. General limitations of using secondary data from NHANES in assessing risk of early kidney dysfunction, including using self-reports, had been previously noted in our earlier study.^11^ For example, the inherent nature of secondary data analysis, including not designing the study specifically for the purpose we evaluated it for is obvious, as is the possibility of recall bias of participants when asked to self-report. Self-reporting a disease state can also be inaccurate. Here, they were asked about SRRD and participants may not know exactly what their condition was, or if it was truly a renal dysfunction. That definition could be subject to interpretation.

Other comorbidities that were not noted here could also have an effect. However, there are other specific limitations applicable to this current analysis. These included the choice of prediction equation for estimating GFR. We resorted to the 2009 CKD-EPI equation, which is based on SCr accounting for physiological contribution of muscle mass estimated through gender, race, and age. The accuracy of this estimating equation has long been contemplated and assessed by the members of the CKD-EPI Collaboration who also developed a 2012 version based on creatinine and cystatin C. In general, equations with multiple endogenous filtration markers are considered more precise than a single filtration marker counterpart.^18,19^ Also, a substantial proportion of the sampled population with reported renal dysfunction or elevated SCr did not report their gender, race, age, education, or income. This greatly reduced the analytical sample and thus statistical power in some situations. No missing data imputation was performed. Subsequent analyses may assess sensitivity of the effect measures related to missing data and indicate potential implications related to these findings.

## Conclusion

Through this assessment of the NHANES dataset, we conclude that there is a significant risk for SRRD and kidney biomarker abnormalities in those consuming labeled APAP use along with LMA consumption. This risk could be greater for women, Hispanics, and blacks more than for whites. Income levels and education levels may have an effect, but this could also be from other unknown effect modifications. Further research could increase the sample size of minority groups and specifically assess for effect modifiers that NHANES does not account for.

## Abbreviations Used

AOR: adjusted odds ratios
APAP: acetaminophen
BP: blood pressure
CHPA: Consumer Healthcare Products Association
CI: confidence interval
CKD-EPI: Kidney Disease–Epidemiology Collaboration
GFR: glomerular filtration rate
LMA: light to moderate alcohol
NHANES: National Health and Nutrition Examination Survey
NPE: national population estimate
OR: odds ratios
SCr: serum creatinine
SRRD: self-reported renal dysfunction
